# Absence of Intraocular Lymphatic Vessels in Uveal Melanomas with Extrascleral Growth

**DOI:** 10.3390/cancers11020228

**Published:** 2019-02-15

**Authors:** Jackelien G. M. van Beek, Quincy C. C. van den Bosch, Nicole Naus, Dion Paridaens, Annelies de Klein, Emine Kiliç, Robert M. Verdijk, on behalf of the Rotterdam Ocular Melanoma Studygroup (ROMS)

**Affiliations:** 1 Department of Ophthalmology, Erasmus University Medical Center, 3015 GD Rotterdam, The Netherlands; n.naus@erasmusmc.nl (N.N.); D.Paridaens@oogziekenhuis.nl (D.P.); e.kilic@erasmusmc.nl (E.K.); 2 Department of Ophthalmology, Albert Schweitzer Hospital, 3318 AT Dordrecht, The Netherlands; 3 Department of Pathology, Erasmus University Medical Center, 3015 GD Rotterdam, The Netherlands; q.vandenbosch@erasmusmc.nl (Q.C.C.v.d.B.); r.verdijk@erasmusmc.nl (R.M.V.); 4 Oculoplastic and Orbital Surgery, Rotterdam Eye Hospital, 3011 BH Rotterdam, The Netherlands; 5 Department of Clinical Genetics, Erasmus University Medical Center, 3015 GD Rotterdam, The Netherlands; a.deklein@erasmusmc.nl

**Keywords:** extrascleral extension, uveal melanoma, lymphatic vessels, LYVE-1, D2-40, Prox-1, CD31, CD34

## Abstract

The aim of this study was to investigate the presence of intraocular lymphatic vessels in patients with uveal melanomas and extrascleral extension using a panel of lymphatic markers. The following immunohistochemical markers were analyzed: lymphatic vessel endothelial hyaluronic acid receptor-1 (LYVE-1), podoplanin (D2-40), prospero-related homeobox gene-1 (Prox-1), pan-endothelial marker cluster of differentiation 31 (CD31), and blood vessel endothelium-specific CD34. Lymphatic vessels were defined as a combination of staining of the following positive markers: LYVE-1, D2-40, Prox-1, and CD31; and no staining of the negative marker CD34. In total, 456 patients were enucleated; 16 of the 46 uveal melanomas with extrascleral extension were contained in stored paraffin tissue. Two samples of the 16 uveal melanomas showed focal positive intraocular vascular staining for LYVE-1 and co-expression of CD31 and CD34. Due to the lack of Prox-1 and D2-40, and positive expression of CD34, these cannot be classified as lymphatic vessels. In one case recruitment of an extraocular, intratumoral lymphatic vascular structure was observed in the periphery of the subconjunctival extrascleral extension. Intraocular lymphatic vessels are absent in uveal melanomas with extrascleral extension; however, we provide proof for recruitment of intratumoral lymphatics by uveal melanomas with extraocular extension from subconjunctival lymphatics that may explain the rare cases of regional lymphatic spread. A panel of antibodies is necessary to detect lymphatic vessels with high specificity.

## 1. Introduction

Ocular melanoma comprises 3% of all melanomas and may originate intraocularly from the uvea or extraocular from the conjunctiva [1]. In contrast to conjunctival melanoma, uveal melanomas (UM) are known to metastasize primarily in a hematogenous manner. Lymphatic metastasis of UM in regional lymph nodes is exceptionally rare and is associated with extraocular extension or orbital recurrence [2,3,4,5]. Extraocular extension occurs in 2–15% of UM and is associated with decreased survival [6]. Various routes for the extraocular extension were described, such as aqueous channels, ciliary arteries, vortex veins, ciliary nerves, optic nerve, and a variety of rare combinations of these routes [7]. Extraocular spread of UM may provide access to subconjunctival lymphatics and, moreover, was hypothesized by some to promote intraocular lymphangiogenesis through recruitment [5]. The lymphatic system is important in providing the products for immune response, interstitial fluid balance, and macromolecular absorption (lipids). The vessel wall consists of the tunica intima, media, and adventitia; however, it is less organized than in blood vessels. There is functional evidence for lymphatic-like tissue in the anterior uvea, and true lymphatic vessels are present in the bulbar conjunctiva [8]. The choroid is known to be alymphatic and whether or not intraocular “classical” lymphatics exist is, therefore, controversial. Several studies suggested that lymphatic vessels are present in the human eye and are supposedly involved in lymphatic metastasis of intraocular malignancies. Lymphatic vessels are derived from venous endothelial cells during embryogenesis. Fetal human sclera is free of lymphatic vessels, but not of blood vessels [9]. Some reported pseudo-lymph vessel appearance in human choroid, while others proposed that intraocular lymphatic vessels with continuous endothelial lining do not exist [8,10]. Tumor-associated ocular lymphangiogenesis was detected in the most common malignant ocular surface tumors of the eye, such as conjunctival carcinoma and melanoma [11,12,13]. Peritumoral intraocular lymphatic vessels were also reported in UM with and without extraocular extension, and it was suggested that intraocular lymphangiogenesis in ciliary body melanomas with extraocular extension may be considered as a new prognostic factor [5,14,15]. However, this depends on which definition is used for a lymphatic vessel.

Lymphatic vessels can be identified using a panel of immunohistochemical markers [16,17,18]. Lymphatic endothelial markers are expressed in lymphatic endothelium, but not in blood vessel endothelium. New endothelial markers recognize growth factors and differentiation antigens specific for lymphatic endothelial cells. It is recommended to use a minimum panel of three endothelial antibodies, which consists of one pan-endothelial marker and at least two different lymphatic endothelial-specific antibodies, to avoid overinterpretation of false staining results [16]. Podoplanin is a mucin-type transmembrane glycoprotein, expressed in lymphatic endothelial cells and other cells such as macrophages and tumor cells [19,20]. Its function includes regulation of lymphatic vascular formation and platelet aggregation. D2-40 is the most commonly used mouse monoclonal antibody against Podoplanin. Prospero-related homeobox gene-1 (Prox-1) is an important lymphatic differentiation factor, a nuclear transcription factor, and of importance for the development of the lymphatic system. Prox-1 is also expressed in nonendothelial cell types, such as hepatocytes, bile duct epithelium, pancreatic epithelium, cardiomyocytes, lens, retina, and spinal and vegetative ganglia [21,22]. It is not expressed in blood vascular endothelial cells, except for a small segment of the embryonic anterior cardinal vein [23]. Knockout models of Prox-1 discovered the crucial role of Prox-1 in the development of the lymphatic system [24]. Lymphatic vessel endothelial hyaluronic acid receptor-1 (LYVE-1) is an integral membrane glycoprotein and lymphatic vessel endothelial hyaluronan receptor type 1. LYVE-1 is expressed in lymphatic, but not in blood vascular endothelium [19]. LYVE-1 may also be expressed by activated macrophages [25]. Cluster of differentiation 31 (CD31; platelet endothelial cell adhesion molecule-1, PECAM) is the most sensitive and specific pan-endothelial marker. It is an integral membrane glycoprotein, expressed on endothelial intercellular junctions, but may also be expressed by macrophages [26]. The marker CD34 is a single-chain transmembrane glycoprotein, a hematopoietic progenitor cell antigen, and is expressed in the endothelial cells of blood vessels, but not in non-neoplastic lymphatic vessels. All these markers may also be expressed in other cells than lymphatic endothelium; therefore, it is difficult to identify lymphatic vessel based on a single marker. When multiple markers are used, lymphatic vessels will be identified with increased accuracy.

Earlier published data suggested the presence of intraocular lymphatic vessels in UM with and without extrascleral extension; however, this was based on restricted immunohistochemical panels [5,14]. Therefore, we analyzed the presence of lymphatic vessels in eyes with UM and extrascleral extension with an extensive panel of lymphatic markers. It is important to interpret the staining pattern with care in order to identify a vascular structure as a lymphatic vessel [16]. In the current study, a lymphatic vessel was identified when it showed expression of the following markers: D2-40, Prox-1, LYVE-1, and CD31, and lacked expression of CD34.

## 2. Results

### 2.1. Patients

In total, 456 uveal melanoma patients were enucleated from 1993 until 2016, of which 417 patients underwent primary enucleation and 39 patients underwent secondary enucleation after fractionated stereotactic radiation therapy. Forty-six tumors showed extrascleral extension [6]. Following the hypothesis that expression of lymphangiogenic growth factors such as vascular endothelial growth factor-c (VEGF-C) in UM may induce secondary lymphangiogenesis only when direct access to pre-existing lymphatic vessels is present, we selected such cases for immunohistochemical investigation [5,27]. Sixteen tumors contained extrascleral tumor extension in the stored paraffin tissue blocks and were selected for immunohistochemical investigation of tumor-associated intraocular lymphatics. For the remaining 30 tumors, tissue with visible extrascleral extension was not available. The mean largest diameter of the extension was 2.6 mm (standard deviation 2.5 mm). Patients and tumor characteristics are shown in Table 1. Fourteen patients underwent primary enucleation. One patient developed untreatable neovascular glaucoma and was treated with secondary enucleation. One patient was exenterated, because, at first presentation, the tumor was too large for enucleation.

### 2.2. Immunohistochemistry

**Figure 1:**
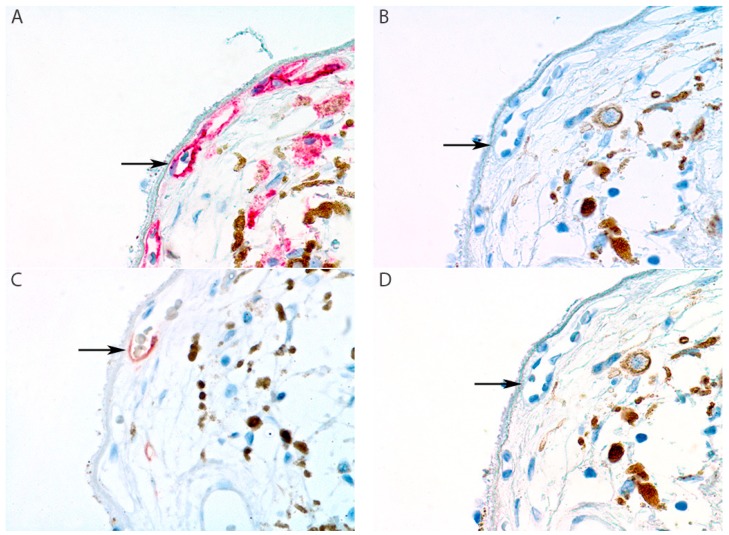

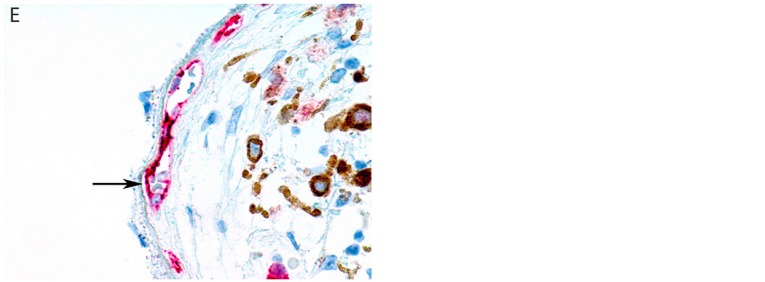
Sample 8 of Table 2: peritumoral focal positive staining of choriocapillary vasculature for lymphatic vessel endothelial hyaluronic acid receptor-1 (LYVE-1). The staining pattern of the five markers are shown (arrows). (**A**) Cluster of differentiation 31 (CD31) stains all endothelial cells. (**B**) Podplanin (D2-40) is negative. (**C**) LYVE-1 shows focal positive staining in a vessel of the choriocapillaris. (**D**) Prospero-related homeobox gene-1 (Prox-1) is negative. (**E**) CD34 stains all endothelial cells. (All panels: original magnification 400×).

**Figure 2:**
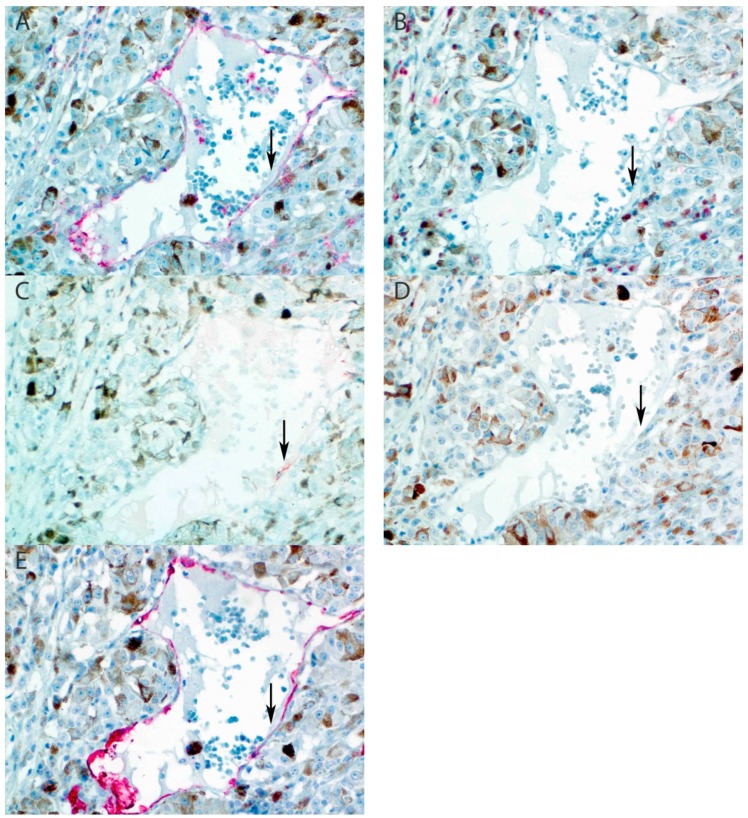
Sample 15 of Table 2: intratumoral focal positive staining of tumor vasculature for LYVE-1. (**A**) CD31 stains all endothelial cells (arrow). (**B**) D2-40 is negative in endothelium. (**C**) LYVE-1 shows focal positive staining in a large tumor vessel. (**D**) Prox-1 is negative. (**E**) CD34 stains all endothelial cells. (All panels: original magnification 400×).

Immunohistochemical analysis showed intraocular peritumoral and intratumoral positive staining for one lymphatic marker in two samples (sample 8 and 15 in Table 2; Figure 1 and Figure 2). However, these vascular structures showed co-expression of CD31 and CD34, and only focal expression of LYVE-1. Due to the lack of Prox-1 and D2-40 expression, these vascular structures cannot be classified as lymphatic vessels. Specifically, we did not find one sample that had an intraocular vascular structure positive for D2-40, Prox-1, LYVE-1, and CD31, with concurrent negative staining for CD34, as in the conjunctival control (Appendix A, Figure A3).

Immunohistochemical analysis showed intraocular peritumoral and intratumoral positive staining for one lymphatic marker in two samples (sample 8 and 15 in Table 2; Figure 1 and Figure 2). However, these vascular structures showed co-expression of CD31 and CD34, and only focal expression of LYVE-1. Due to the lack of Prox-1 and D2-40 expression, these vascular structures cannot be classified as lymphatic vessels. Specifically, we did not find one sample that had an intraocular vascular structure positive for D2-40, Prox-1, LYVE-1, and CD31, with concurrent negative staining for CD34, as in the conjunctival control (Appendix A, Figure A3).

**Figure 3:**
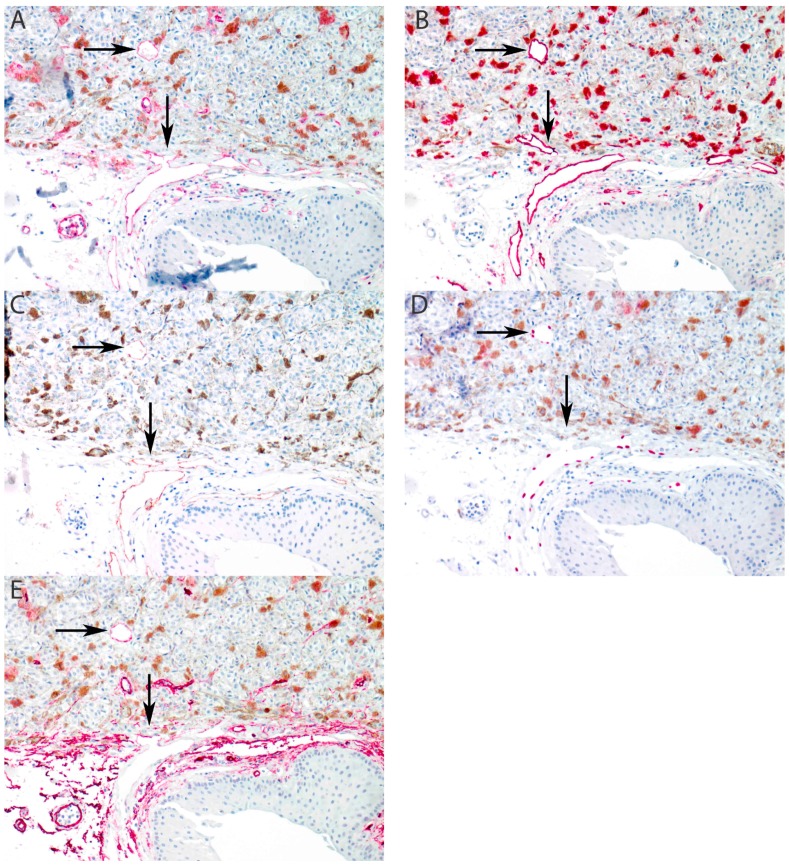
Recruitment of lymphatic vessels into extraocular extension of uveal melanoma (arrows). (**A**) CD31 stains all endothelial cells. (**B**) D2-40 stains conjunctival lymphatic vessel endothelium and demonstrates intratumoral recruitment. (**C**) LYVE-1 stains conjunctival lymphatic vessel endothelium and demonstrates intratumoral recruitment. (**D**) Prox-1 is positive in the nuclei of lymphatic endothelial cells and demonstrates intratumoral recruitment. (**E**) CD34 is positive in blood vessel endothelium and negative in lymphatic endothelium. Note that the intratumoral recruited lymphatic vessel stains weakly positive at the recruitment front (vertical arrow) and intatumoral (horizontal arrow). (All panels: original magnification 100×).

**Figure A1:**
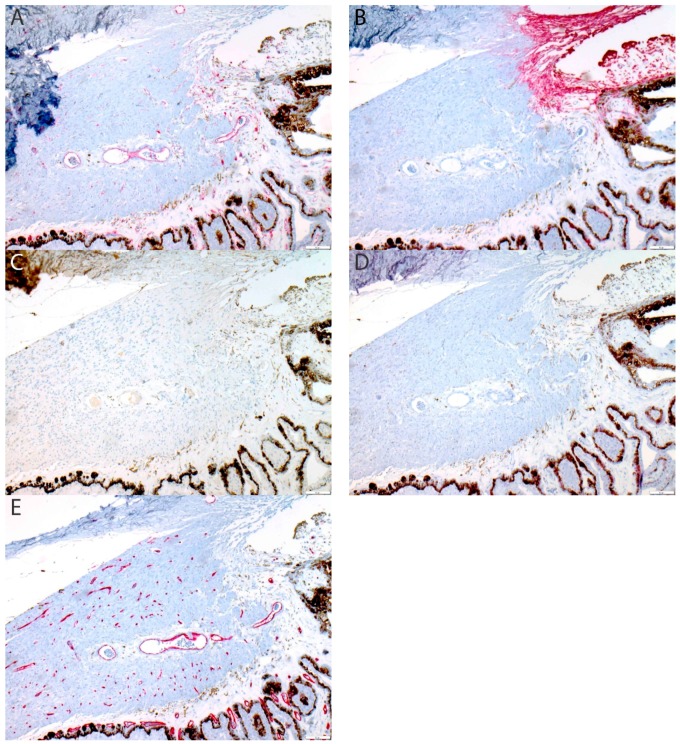
Positive staining of the trabecular meshwork, iris, and anterior ciliary body for podoplanin (D2-40) in an eye without uveal melanoma. (**A**) CD31 stains all vascular endothelial cells and shows focal positivity in macrophages. (**B**) D2-40 stains the stroma of the trabecular mesh work, iris, and anterior ciliary body and shows focal positivity in macrophages. (**C**) LYVE-1 is negative. (**D**) Prox-1 is negative. (**E**) CD34 stains all vascular endothelial cells. (All panels: original magnification 25×).

**Figure A2:**
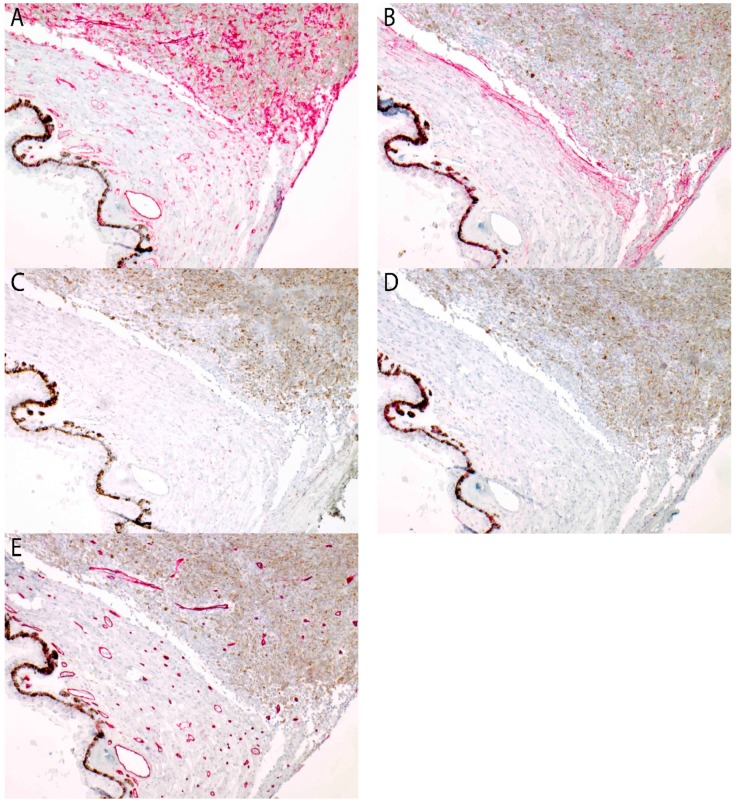
Positive staining of the anterior ciliary body for D2-40 in uveal melanoma. (**A**) CD31 stains all vascular endothelial cells. (**B**) D2-40 stains the peritumoral stroma of the anterior ciliary body. (**C**) LYVE-1 is negative. (**D**) Prox-1 is negative. (**E**) CD34 stains all vascular endothelial cells. (All panels: original magnification 50×).

**Figure A3:**
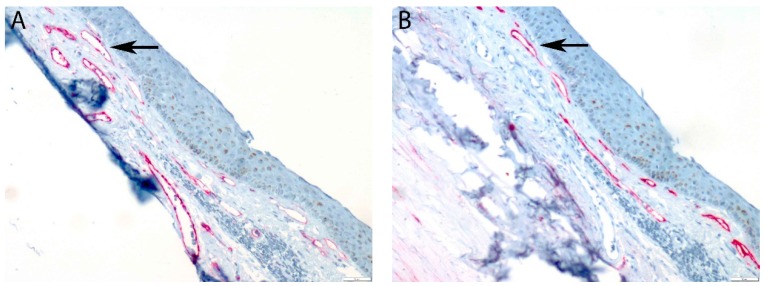

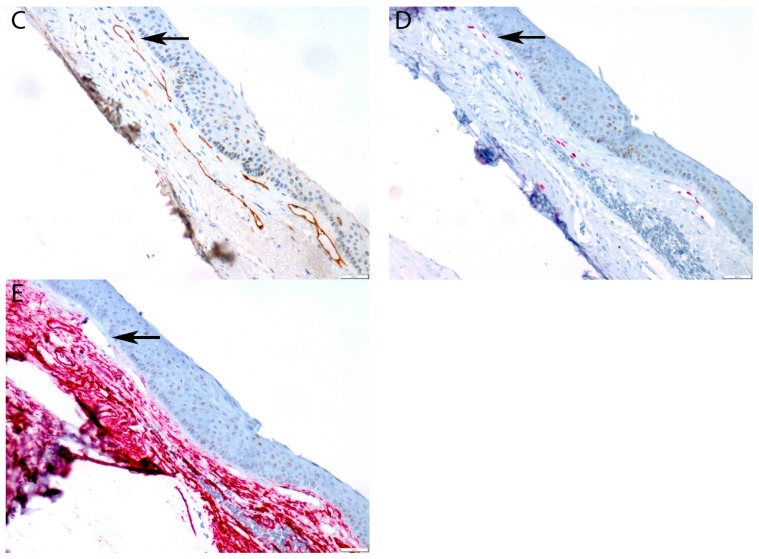
Conjunctival control tissue staining pattern. (**A**) CD31 stains all endothelial cells (arrow). (**B**) D2-40 stains conjunctival lymphatic vessel endothelium (arrow). (**C**) LYVE-1 stains conjunctival lymphatic vessel endothelium (arrow). (**D**) Prox-1 is positive in the nuclei of lymphatic endothelial cells (arrow). (**E**) CD34 is positive in blood vessel endothelium and negative in lymphatic endothelium (arrow). Conjunctival stroma also stains positive. (All panels: original magnification 100×).

We paid special attention to conjunctival lymphatic vessel recruitment in cases of anterior extrascleral extension of ciliary body melanomas. In one case (in addition to the two samples mentioned earlier), without showing any of intraocular lymphatic markers, an extraocular, intratumoral lymphatic vascular structure was observed in the periphery of the extrascleral extension of the tumor. However, no intraocular recruitment was observed in this case (Figure 3). Positive staining for D2-40 was observed in the trabecular meshwork and anterior ciliary body of eyes without UM as reported before (Appendix A, Figure A1), as well as in cases of ciliary body melanoma (Appendix A, Figure A2).

## 3. Discussion

This study, with an extensive panel of immunohistochemical markers, examined the presence of intraocular lymphatic vessels and lymphatic vessels adjacent to the intratumoral part of the extrascleral extension of UM. Our panel of markers made it possible to distinguish lymphatic vessels with certainty from blood vessels. We could not detect intraocular lymphatic vasculature in UM samples with extraocular extension using these five specific markers. However, we did find two samples with intraocular focal vascular positivity for LYVE-1, but negative staining for Prox-1 and D2-40. Moreover, these vascular structures were positive for CD34. These two samples had T2 and T4 tumors; both had mixed cell types and were choroid and ciliary body UM, respectively (see Figure 1 and Figure 2).

Our findings do not support the findings by Heindl et al., who detected intraocular LYVE-1 (+) and podoplanin (+) lymphatic vessels in 12 out of 20 ciliary body melanoma with extraocular extension and associated this with worse prognosis [5]. In the extraocular tumor component, all tumors of that study showed subconjunctival peritumoral vessels that were LYVE-1 (+) and podoplanin (+), but notably no intratumoral recruitment was observed. These authors only used lymphatic markers and did not evaluate co-expression of vascular markers such as CD31 or CD34 in these proposed lymphatic vascular structures, as would be required by the consensus statement. This implies that the functionality of these structures as lymphatic vessels remains controversial, as was also emphasized by the authors [5]. Although our findings are in concordance with Khan et al., who described peritumoral staining for the lymphatic marker podoplanin in UM with ciliary body involvement with or without extraocular extension, we draw a different conclusion [14]. In that study, supposed lymphatic vessels were identified with D2-40 in combination with negative staining for the blood vessel marker CD34. This study also described possible lymphatic structures in the ciliary body in eyes without UM. The staining pattern described in this study was commented on in a letter to the editor by Heindl et. al. who rightly argued that this staining pattern merely represented earlier described endothelial marker expression in the anterior segment of the human eye [8,28]. We are able to support this commentary by our staining results in tumor tissue and non-tumor tissue specimens. In addition, we observed peritumoral positive staining of the ciliary body and choroidal stroma by D2-40 without co-expression of LYVE-1 or Prox-1. D2-40 is, just as LYVE-1, expressed in lymphatic endothelial cells, but also in various other cells such as macrophages and in various structures in the anterior segment of the eye. This makes it important to use more than just one immunohistochemical lymphatic vessel marker. Multiple consensus statements advise, among others, a panel of at least two lymphatic markers and one vascular marker for identification of lymphatics [16,18].

Other studies support our observations and did not observe any intraocular lymphatic vessels in uveal melanoma. Furthermore, although other markers were used, Clarijs et al. found, with the presence of pan-endothelial CD31, lymphatic endothelium specific Flt-4 (Fms related tyrosine kinase 4), and blood vessel endothelial marker CD34 expression, no sufficient evidence that lymphangiongenesis was induced from preexisting blood vessels in UM [27]. Within the eye “atypical” lymphatic marker expression might exist, such as endothelial cells of Schlemm’s canal that tend to show a positive expression of Prox-1 [29]. On the other hand, some lymphatic vessel function was demonstrated in special vascular structures of the eye, such as Schlemm’s canal [30]. However, these cells do not show all features of terminally differentiated lymphatic endothelial cells. Moreover, the human choroid is endowed with a significant number of LYVE-1 (+) macrophages [10].

The tumor, node, metastasis (TNM) classification of UM is based on the extent of the primary tumor and on the presence of any systemic metastases, since lymph node involvement is extremely rare [31]. It is well known that UM may metastasize to the liver (and elsewhere in the body) through hematogenous spread. Only a few cases of patients with UM and extraocular spread with regional lymph node metastasis were described. In two cases with extraocular spread, regional lymph node metastases were found, and Heindl et al. reported regional lymph node metastasis in 3/20 patients with extraocular spread [3,5]. Ardjomand et al. described lymph node metastases from a ciliary body ring melanoma upon repeated trabeculectomy [4]. Tojo et al. reported 5/77 patients who developed cervical metastases associated with prior orbital recurrence and distant metastatic disease [2]. Our study is the first to illustrate recruitment of subconjunctival lymphatics into the extraocular extension in a case of ciliary body melanoma. This finding may indeed explain regional lymphatic spread of UM by way of access of uveal melanoma cells to the subconjunctival lymphatics through (iatrogenic) extrascleral spread of the tumor. However, no regional lymphatic metastasis was documented in our patient. It must be noted that the intratumoral recruited lymphatic structure expresses uneven staining for LYVE-1, when compared to the conjunctival lymphatic vessel, and shows weak expression of CD34. This indicates the need for further functional studies of such recruited vessels.

Macrophages were suggested to stimulate neo-lymphangiogenesis in settings of inflammation. Bone-marrow-derived CD11b+ macrophages expressed lymphatic endothelial markers such as LYVE-1 and Prox-1 under inflamed conditions in the corneal stroma of mice. In vitro experiments demonstrated that CD11b+ macrophages alone were capable of forming tube-like structures that expressed markers of lymphatic endothelium such as LYVE-1 and podoplanin [32]. Uveal melanoma may show increased numbers of CD11b+ macrophages [33]. CD31, D2-40, and LYVE-1 were found to be positive in tumor-associated macrophages. In addition, vessels in a UM or near a UM could be focally positive for LYVE-1. However, when all other lymphatic markers are negative and CD34 is positive, these structures cannot be classified as functional lymphatic vessels. LYVE-1 may be positive in non-endothelial cells, as mentioned earlier. Therefore, the earlier described positive staining could easily be seen as positive staining of the macrophages, which are normally present in the choroid. When less than these five markers are used, false positive interpretation of the staining results could be passed for proof of lymphatic vessels. This is the reason why we prefer to use more markers than already advised. Functional studies of such aberrant staining vascular structures are needed to further substantiate our point.

## 4. Materials and Methods

### 4.1. Sample Selection

Eyes of patients with a choroidal or ciliary body UM with extraocular extension, who underwent primary or secondary enucleation, were selected from the Rotterdam Ocular Melanoma Studygroup (ROMS) database from 1993 until 2016 [6]. Sample size was not determined statistically in advance. Patients with iris melanoma and patients without a pathology report, describing the histopathological characteristics of the tumor, were excluded. Tumor characteristics, the largest tumor diameter and the tumor thickness, were measured on B-scan ultrasonography and, if those results were unknown, we used the measurements from the pathology report. This was also done for the diameter of the extraocular extension. Until 1999, all patients were enucleated; hereafter, enucleation was only performed if the tumor was too large for fractionated stereotactic radiotherapy (basal largest tumor diameter >16 mm and tumor thickness >12 mm) or if the patient requested enucleation. Disease-free survival was defined as the moment of diagnosis until development of metastases or patient death. Cases in which the cause of death was unknown or not related to UM were treated as censored. Survival data was obtained until July 2017. The oncologist determined if there were metastases, and patients received routine blood tests during follow-up or ultrasound of the liver. Informed consent was obtained prior to treatment, and the study was performed according to guidelines of the Declaration of Helsinki [34]. The Medical Ethics Committee of the Erasmus MC approved this study (MEC-2009-375).

### 4.2. Tissue Processing

Fresh tumor material was obtained within one hour of enucleation, and the globes were processed for further histopathological analysis. Conventional histopathologic examination with hematoxylin and eosin (H&E) staining of formalin-fixed and paraffin-embedded (FFPE) eyes was performed on all tumors and confirmed the origin of the tumor, presence of inflammation, and necrosis. H&E staining was used to differentiate between an epithelioid, mixed, or spindle cell type according to the modified Callender classification [35]. Extraocular extension was defined as tumor growth through the sclera and beyond the outer scleral surface. Subsequently, the largest diameter of the extension of the tumor on the sclera surface was measured. The orbital fat resection margin was examined for presence of malignant cells. We determined the route of extraocular spread and involvement of optic nerve, ciliary body, or choroid.

### 4.3. Immunohistochemistry

FFPE tumor tissue of uveal melanomas with extrascleral extension of 46 patients was analyzed for the presence of lymphatic vessels. Four-micrometer-thick sections were stained for podoplanin (Clone D2-40, reference no. 760-4395, Cell Marque, Rocklin, CA, USA), prospero homeobox-1 (Prox-1, Clone D2J6J, dilution 1:1500, Cell signaling, Leiden, The Netherlands), cluster of differentiation 31 (CD31, Clone JC70, reference no. 760-4378, Cell Marque, Rocklin, CA, USA), and cluster of differentiation 34 (CD34, Clone QBEnd/10, reference no. 790-2927, Ventana, Tucson, AZ, USA) with the Ventana Benchmark Ultra automated staining system (Ventana Medical Systems, Tucson, AZ, USA). Briefly, after deparaffinization, the sections were processed for 32–64-minute antigen retrieval using Cell Conditioning Solution 1 (CC1 Ventana reference no. 950-124). Following 32-minute incubation (16-minute for CD31) with the primary antibody at 36 °C, detection was performed using the ultraView Universal Alkaline Phosphatase Red Detection Kit (Ventana reference no. 760-501) in combination with the Amplification Kit (Ventana Ref.: 760-080). Sections were counterstained with hematoxylin II (Ventana reference no. 790-2208). For anti-LYVE-1 (Clone AF2089, dilution 1:1000, R&D Systems, Minneapolis, MN, USA), primary antibody staining was executed using the Ventana Discovery Benchmark automated staining system (Ventana Medical Systems, Tucson, AZ, USA). The following adaptations from the protocol were required: endogenous peroxidase was blocked using Inhibitor CM from the DISCOVERY ChromoMap DAB Kit (RUO) (Ventana, reference no.: 760-159) for four minutes. The secondary antibody incubation was performed with anti-Goat-horseradish peroxidase (HRP; Ventana reference no. 760-159) for 32 minutes. Detection was executed manually with 3-amino-9-ethylcarbazoledue (AEC) diluted in 0.2 M sodium acetate with H_2_O_2_. The slides were counterstained with Mayer’s hematoxylin (Klinipath, Cat. 4085.9005, Duiven, The Netherlands).

### 4.4. Scoring of Immunohistochemistry

Three independent reviewers scored the slides: a pathologist, an ophthalmologist, and a research technician with ample experience in ophthalmic pathology. Lymphatic vessels were identified using a panel of immunohistochemical markers. For this study, a vascular structure was identified as a lymphatic vessel when it displayed an expression of podoplanin, Prox-1, LYVE-1, and CD31 and no expression of CD34. These requirements are in accordance with the first international consensus on the methodology of lymphangiogenesis quantification in solid human tumors [16]. Lymphatic vessels of the perilimbal conjunctiva served as an internal control. External control tissue was properly applied, such as lymphatic tissue of the testis. Expression of the markers in control eyes without UM was evaluated as well.

## 5. Conclusions

We were not able to confirm intraocular lymphangiogenesis in UM, but we do provide support to the possibility of regional lymphatic spread of UM with extraocular extension through subconjunctival lymphatics. We propose a panel of antibodies (LYVE-1, Prox-1, D2-40, CD34, and CD31) to detect intraocular lymphatic vessels with high specificity. Only when the antibodies LYVE-1, Prox-1, D2-40, and CD31 show positive staining combined with negative expression of CD34 can a vascular structure be classified as a lymphatic vessel.

## Figures and Tables

**Table 1:** Baseline patient characteristics.

Patient Characteristics	Patients, *n* = 16
Gender, No. (%)	
Men	7 (44)
Women	9 (56)
Age in years, mean (SD) ^1^	66 (14)
Tumor size classification	
T1	3
T2	3
T3	7
T4	3
Largest diameter of the extension of the tumor in mm, mean (SD) ^1^	2.6 (2.5)
Tumor location	
Choroid	9
Ciliary body	7
Cell type	
Epithelioid	4
Mixed	7
Spindle	5
Disease-free survival in months, mean (SD) ^1^	77 (64)
Alive, *n*	9
Metastases, *n*	4
Death, because of uveal melanoma, *n*	3
Death other cause, *n*	3
Lost to follow-up, *n*	1

^1^ SD: standard deviation.

**Table 2:** Required expression profile of lymphatic vessels and patient samples. CD—cluster of differentiation; D2-40—podoplanin; LYVE-1—lymphatic vessel endothelial hyaluronic acid receptor-1; Prox-1—prospero-related homeobox gene-1.

Markers	Expression of CD31	Expression of D2-40	Expression of LYVE-1	Expression of Prox-1	Expression of CD34
Lymphatic vessel	+	+	+	+	−
Sample 1	+	−	−	−	+
Sample 2	+	−	−	−	+
Sample 3	+	−	−	−	+
Sample 4	+	−	−	−	+
Sample 5	+	−	−	−	+
Sample 6	+	−	−	−	+
Sample 7	+	−	−	−	+
Sample 8	+	−	+	−	+
Sample 9	+	−	−	−	+
Sample 10	+	−	−	−	+
Sample 11	+	−	−	−	+
Sample 12	+	−	−	−	+
Sample 13	+	−	−	−	+
Sample 14	+	−	−	−	+
Sample 15	+	−	+	−	+
Sample 16	+	−	−	−	+
